# Gut proinflammatory bacteria is associated with abnormal functional connectivity of hippocampus in unmedicated patients with major depressive disorder

**DOI:** 10.1038/s41398-024-03012-9

**Published:** 2024-07-16

**Authors:** Shu Xiao, Zibin Yang, Hong Yan, Guanmao Chen, Shuming Zhong, Pan Chen, Hui Zhong, Hengwen Yang, Yanbin Jia, Zhinan Yin, Jiaying Gong, Li Huang, Ying Wang

**Affiliations:** 1 Medical Imaging Center, First Affiliated Hospital of Jinan University, Guangzhou, China; 2 Institute of Molecular and Functional Imaging, Jinan University, Guangzhou, China; 3 Department of Psychiatry, First Affiliated Hospital of Jinan University, Guangzhou, China; 4 Biomedical Translational Research Institute, Jinan University, 510630 Guangzhou, China; 5 Department of Radiology, Six Affiliated Hospital of Sun Yat-sen University, Guangzhou, China

**Keywords:** Depression, Diagnostic markers

## Abstract

Accumulating evidence has revealed the gut bacteria dysbiosis and brain hippocampal functional and structural alterations in major depressive disorder (MDD). However, the potential relationship between the gut microbiota and hippocampal function alterations in patients with MDD is still very limited. Data of resting-state functional magnetic resonance imaging were acquired from 44 unmedicated MDD patients and 42 demographically matched healthy controls (HCs). Severn pairs of hippocampus subregions (the bilateral cornu ammonis [CA1-CA3], dentate gyrus (DG), entorhinal cortex, hippocampal–amygdaloid transition area, and subiculum) were selected as the seeds in the functional connectivity (FC) analysis. Additionally, fecal samples of participants were collected and 16S rDNA amplicon sequencing was used to identify the altered relative abundance of gut microbiota. Then, association analysis was conducted to investigate the potential relationships between the abnormal hippocampal subregions FC and microbiome features. Also, the altered hippocampal subregion FC values and gut microbiota levels were used as features separately or together in the support vector machine models distinguishing the MDD patients and HCs. Compared with HCs, patients with MDD exhibited increased FC between the left hippocampus (CA2, CA3 and DG) and right hippocampus (CA2 and CA3), and decreased FC between the right hippocampal CA3 and bilateral posterior cingulate cortex. In addition, we found that the level of proinflammatory bacteria (i.e., *Enterobacteriaceae*) was significantly increased, whereas the level of short-chain fatty acids producing-bacteria (i.e., *Prevotellaceae, Agathobacter* and *Clostridium*) were significantly decreased in MDD patients. Furthermore, FC values of the left hippocampal CA3- right hippocampus (CA2 and CA3) was positively correlated with the relative abundance of *Enterobacteriaceae* in patients with MDD. Moreover, altered hippocampal FC patterns and gut microbiota level were considered in combination, the best discrimination was obtained (AUC = 0.92). These findings may provide insights into the potential role of gut microbiota in the underlying neuropathology of MDD patients.

## Introduction

Major depressive disorder (MDD) is a serious and chronic psychiatric disease, and remains one of the most important worldwide causes of disability, morbidity, and mortality from suicide [1]. The core symptoms of MDD are characterized by persistently depressed mood and/or loss of pleasure or overall interest in life [2], frequently accompanied by functional and cognitive impairment that serious impact on social functioning and quality of life of patients [3]. Converging evidence indicates that the gut microbiota could bidirectionally communicate with the central nervous system through several mechanistic pathways (e.g., immunoregulatory, nervous, or metabolic pathways) [4–6], which comprise the “gut-brain axis” (GBA), thereby influencing host emotional regulation, cognition and behavior [7, 8]. Despite dysregulated GBA of MDD have been reported in previous studies [7, 9], the relationship between brain function and gut microbiota alterations in MDD remains unclear.

Accumulating studies have revealed that the hippocampus may be a core region of the neural physiopathology of MDD [10, 11]. Data from postmortem studies have found decreased neuronal number, decreased spine density, and increased neuro-inflammatory gene expression in hippocampus in patients with MDD [12, 13]. In addition, structural and functional alterations of the hippocampus were also observed in neuroimaging studies of MDD, including reduced gray matter volume [14, 15], impaired white matter integrity [16], and abnormal spontaneous brain activity [15, 17]. The hippocampus is a core region of the limbic system that has an essential role in multiple cognitive functions (especially including learning and memory acquisition and consolidation, as well as declarative memory retrieval) and emotion regulation, neuroendocrine stress response and motivational behaviors [18–23]. However, the hippocampus is not a uniform structure but consists of multiple subfields with distinct functions [20, 23], which could be divided into 7 distinct subregions: the cornu ammonis (CA1-CA3), dentate gyrus (DG), subiculum (Subc), entorhinal cortex (EC), and hippocampal–amygdaloid transition area (HATA) [24]. Within the hippocampus, the CA1-CA3 are involved in learning and memory functions, which mainly participates in short-term spatial and contextual memory [25]; the DG is related in pattern separation of processing novel information [26]; the Subc is the core components of hippocampal information output and also the effector of the baroreceptor reflex [27]; and the EC and HATA are associated with emotional modulation and emotional memory [28]. Most of previous resting-state functional magnetic resonance imaging (rs-fMRI) studies investigated functional connectivity (FC) alterations in adult MDD using the whole hippocampus as the seed region [29–31]. Only few studies have explored FC alterations of hippocampal subregions in adult MDD. Hao et al. [32] found increased FC between the bilateral hippocampus (mainly CA regions) and between the hippocampal CA regions and right insula in patients with MDD. Song et al. [33] revealed decreased FC between the right hippocampal Subc and right middle frontal gyrus in MDD patients with anti-depressant treatment. Wang et al. [34] demonstrated greater changes in the FC between the left hippocampal CA and bilateral posterior cingulate cortex (PCC) in remitted late-onset depression. The inconsistency might be explained by age ranges, different clinical statuses of MDD (i.e., first-episode, medicated, and remitted), and methodology. However, these studies failed to explore finer divisions of hippocampus in rs-FC studies in adult unmedicated MDD.

A growing body of literature suggest that gut microbiome dysfunction is significantly involved in the pathogenesis of MDD. Recent studies have suggested that inflammatory bacteria are a series of bacteria that could regulate inflammation by influencing differentiation of inflammatory cell types, cytokine production and hematopoiesis, such as *Enterobacteriaceae*, *Escherich, Prevotalla* and *Clostridium*[35, 36]. Previous systematic review meta-analyses reported increased relative abundance of proinflammatory bacteria (*Enterobacteriaceae* and *Escherich*), and decreased relative abundance of probiotics (*Faecalibacterium*, *Prevotalla*, *Clostridium*, and *Ruminococcus*) in patients with MDD [9, 37]. Accumulating evidence has suggested that the intestinal microbiota has a crucial role in the functioning of the nervous system, especially involvement in the development of the hippocampus in MDD [38, 39]. Furthermore, research in mice with fecal transplantations from patients with MDD has exhibited depression-like behavior and displayed significant disturbances of the products of probiotic metabolism (such as short-chain fatty acids [SCFAs], lactic acid, and amino acid) [40, 41], which may further contribute to the inflammatory response [42]. Another review showed that over-expression of hippocampal nuclear receptors (activated by microbiota metabolites) could cause depression-like symptoms and decreased expression of brain derived neurotfrophic factor (BDNF) in the hippocampus in naïve rats [43]. However, there has no studies have revealed the potential relationship between the gut microbiota and brain function alterations, especially in the hippocampus, in patients with MDD.

Recently, many neuroimaging studies have applied machine learning methods to classifying patients with MDD and healthy controls (HCs) [44, 45]. Support vector machine (SVM), a multivariable pattern recognition technology, has good classification effect in dealing with nonlinear, high-dimensional and small sample data sets. The SVM has good performance in finding the optimal separation hyperplane for clinical diagnosis. Many studies reported good classification between MDD patients and HCs based on MRI data, however, no studies have combined the features of neuroimaging data and gut microbiota composition.

In this study, we herein aimed to investigate relationship between the FC alterations of hippocampal subregions and gut microbiota composition in MDD patients, and use the SVM method to classify MDD patients and HCs based on the features of altered FC patterns of hippocampus and gut microbiota composition, which may provide new insights and objective neuroimaging evidence to elucidate the potential neuropathological mechanism of the GBA involved in MDD. Based on previous studies, we hypothesized that abnormal FC patterns of hippocampal subregions may be showed between the regions of the bilateral hippocampus, and between hippocampal subregions and cingulate cortex in patients with MDD. The levels of proinflammatory bacteria may higher, while probiotics lower in MDD. In addition, the hippocampus dysfunction is linked to the gut microbiome dysbiosis in MDD.

## Methods

### Participants

A total of 49 right-handed, adult patients with MDD were recruited from the psychiatry department, first Affiliated Hospital of Jinan University, Guangzhou, China. The participants were aged 18-55 years and were diagnosed with nonpsychotic MDD by two experienced psychiatrists (Y.J. and S.Z., with 22 and 7 years of experience in clinical psychiatry, respectively) based on the DSM-V criteria (Diagnostic and Statistical Manual of Mental Disorders, Fifth Edition), patient version (SCID-I/P). The clinical state of each patient was assessed using the 24-item Hamilton Depression Rating Scale (HDRS) during the 3-day period before the imaging scanning. The MDD patients were suffering from depression (total HDRS-24 score > 21 and Young Mania Rating Scale [YMRS] score < 7). Patients with MDD were excluded in our study if they have (1) other Axis-I psychiatric disorders or (2) a history of electroconvulsive therapy, organic brain disorder, neurological disorders, mental retardation, alcohol/substance abuse, pregnancy, cardiovascular diseases or presence of a concurrent and major physical illness. At the time of scanning, all patients were either medication-naïve, or had been unmedicated for at least 6 months. In addition, 44 right-handed HCs were recruited through local advertisements. They were carefully screened through a diagnostic interview, the SCID Nonpatient Edition, to rule out the presence of the current or past history of any psychiatric illness. Further exclusion criteria for HCs were any history of psychiatric illness in first-degree relatives, current or past significant medical or neurological illness. All the participants did not take any antibiotics, immunosuppressive drugs, probiotics or catharsis drugs for at least two weeks before the examination.

This study was reviewed and approved by the Ethics Committee of First Affiliated Hospital of Jinan University (Guangzhou, China), and the authors assert that all procedures contributing to this work comply with the ethical standards of the relevant national and institutional committees on human experimentation and with the Helsinki Declaration of 1975, as revised in 2008. All participants signed a written informed consent form after a full-written and verbal explanation of the study. Shu Xiao and Zibin Yang was blinded to the group allocation during the experiment and/or when assessing the outcome. Two senior clinical psychiatrists confirmed that all subjects had the ability to consent to participate in the examination.

### Fecal samples collection, DNA extraction, intestinal flora 16S ribosomal DNA gene sequencing and analysis

Fecal samples of subjects were collected within 3 days before or after MR examination, and no obvious diarrhea and constipation was observed in all subjects. To avoid surface contamination, we obtained the fresh middle section of the stool sample, and stored at -80°C immediately. According to the manufacturer’s instructions, the microbial community DNA was extracted using MagPure Stool DNA KF kit B (Magen, China). Variable regions V4 of 16 s rRNA gene was selected for PCR amplification based on the Illumina MiSeq platform and following the Illumina protocol. Clean reads were generated after removing adaptor sequences, short length, ambiguous bases and low complexity [46]. Vsearch was used for joining of paired-end reads, quality control and dereplication. Subsequently, the quality-filtered sequences were mapped to the chimera-free amplicon sequence variants (ASVs) by Usearch [47]. ASVs were obtained as a reference for quantification through alignment using Vsearch. The taxonomy of ASVs was classified with the Ribosomal Database Project (RDP, Version 16) reference database with a minimum confidence threshold of 0.8. Analysis of alpha diversity (abundance-based coverage estimator [48], shannon and simpson indices) [48] and beta diversity (principal coordinate analysis [PCoA] based on Bray-Curtis distances) was conducted based on output normalized data within RStudio software (version 4.1). Furthermore, we applied LEfSe analysis to identify differentially abundant bacterial taxa between MDD and HCs. Only those taxa that obtained a log linear discriminant analysis (LDA) score >2.0, and *p* < 0.05 in Wilcoxon test were ultimately considered statistically different between groups.

### MRI data acquisition and preprocessing

All brain imaging data were acquired by using a GE Discovery MR750 3.0 T System with an 8-channel phased-array head coil. During the scanning, participants were instructed to relax and keep their eyes closed but not to fall asleep, keep motionless as much as possible. Each participant was confirmed to stay awake after the experiment. The rs-fMRI data were obtained using a gradient-echo echo-planar imaging sequence with the following parameters: time repetition (TR)/time echo (TE) = 2000/25 ms; flip angle = 90°; voxel size = 3.75 × 3.75 × 3 mm^3^; field of view (FOV) = 240 × 240 mm2; matrix = 64 × 64; slice thickness/ gap = 3.0/1.0 mm; 35 axial slices covering the whole brain; and 210 volumes acquired in 7 min. In addition, a three-dimensional brain volume imaging (3D-BRAVO) sequence covering the whole brain was used for structural data acquisition with the following parameters: TR/TE = 8.2/3.2 ms; flip angle = 12°; bandwidth = 31.25 Hz; slice thickness/gap = 1.0/0 mm; matrix = 256 × 256; FOV = 240 × 240 mm; NEX = 1; and acquisition time = 3 min 45 s. Routine MRI examination images were also collected to exclude any anatomic abnormality. All MRI data were assessed by two experienced neuroradiologists (Z.Q. and Z.L., with 8 and 6 years of experience in neuroimaging, respectively) to confirm the absence of any brain structural abnormalities.

### Functional image preprocessing

Preprocessing of imaging data was performed using the Data Processing Assistant for Resting-State fMRI (DPABI_V3.0, http://restfmri.net/forum/DPABI) toolbox, which is based on Statistical Parametric Mapping (SPM12, https://www.fil.ion.ucl.ac.uk/spm/) software [49]. The first 10 images of the rs-fMRI data were excluded for each participant to ensure steady-state longitudinal magnetization. The remaining 200 images were first slice-time corrected and then were realigned to the first image for correcting for inter-TR head motion. This realignment correction provided a record of the head motion within the rs-fMRI scan. All participants should have no more than 2 mm maximum displacement in any plane, 2° of angular motion as well as 0.2 mm in the mean frame-wise displacement (FD) [50]. Individual T1 structural images were segmented into 3 parts (i.e., white matter, gray matter, and cerebrospinal fluid) using the segmentation toolbox. Then, the DARTEL toolbox was used to create a study-specific template for accurate normalization. Next, the resting-state functional images were co-registered to the structural images and transformed into the standard Montreal Neurological Institute (MNI) space, resliced to a voxel size of 3 × 3 × 3 mm³ resolution and smoothened using a 6 mm full width at half maximum (FWHM) Gaussian kernel. The data were removed the linear trend and passed through the band-pass filter of 0.01–0.1 Hz. Finally, several nuisance covariates and their temporal derivatives were regressed out from each voxel’s time course, including the signals of the brain global mean, white matter, and cerebrospinal fluid, as well as the Friston-24 parameters of head motion.

### FC analysis of hippocampus and hippocampal subregions

**Fig. 1:**
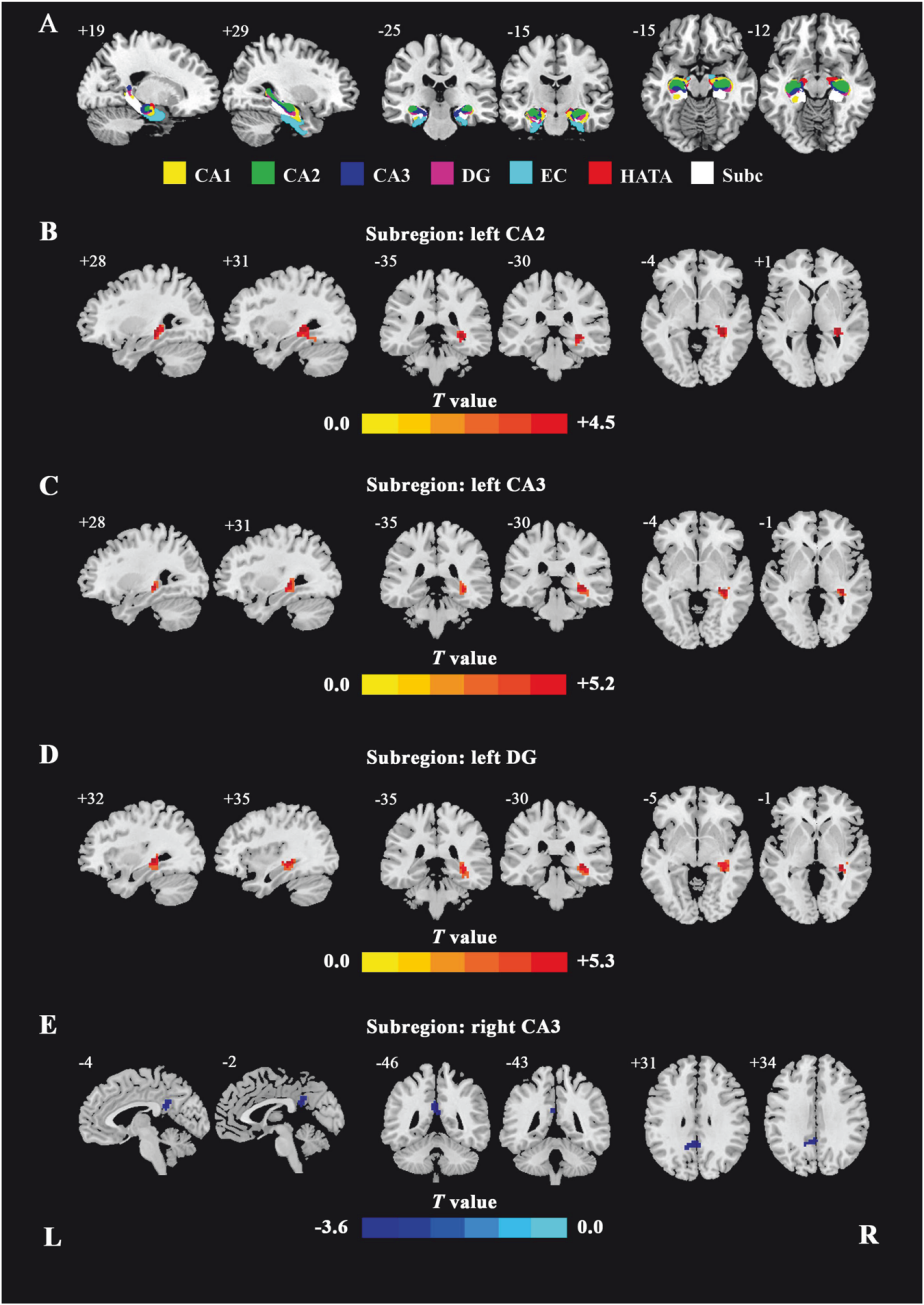
The hippocampal subregions and significant FC differences between the MDD and HCs. **A** The subregions of hippocampus. **B**–**E** Significant FC differences between the MDD and HCs for hippocampal subregion, respectively (voxel *p* < 0.005, cluster *p* < 0.007, GRF corrected). The color bar indicates the *t* values from two-sample *t* test analysis. FC functional connectivity, GRF Gaussian random field, L (R) left (right) hemisphere.

Following previous studies [51], seed-based FC analyses were performed using the DPABI. For the segmentation of the hippocampus’s subregion, we used the template from the Anatomy toolbox’s cytoarchitectonic probabilistic maps, which were originally created from 10 post-mortem brains [24, 52]. Moreover, for each subject, the preprocessed data were registered to MNI standard space (created from 152 individual Brains) [53], and the masks of hippocampus for group FC analysis were also extracted based on MNI standard space. Each hippocampus was segmented into 7 subregions: CA1, CA2, CA3, DG, EC, HATA, and Subc (Fig. 1A). Seed-based FC analyses were conducted for the left and right whole hippocampus and their subfields separately (8 paired seeds in total including bilateral hippocampus, CA1, CA2, CA3, DG, EC, HATA, and Subc) using the DPABI toolbox. To generate individual rs-FC maps, bivariate correlations were calculated between the mean time series of each seed masks and the time series of each voxel in the whole brain. The subject-level correlation maps were converted into z-value maps by Fisher’s r-to-z transform to improve the normality. For all the subjects, 14 z-score maps that represent the intrinsic FC of the 14 hippocampus masks were finally obtained.

### SVM classification

Machine-learning analyses were preformed using the libsvm toolbox version 3.25 (https://www.csie.ntu.edu.tw/~cjlin/libsvm/) in MATLAB2014b. Briefly, a linear SVM model is a hyperplane separating two distinct classes of features (i.e., data from hippocampal FC patterns and gut microbiota) in the most optimal manner. The SVM classification process consists of two steps: training and testing. We selected the radial basis function (RBF) kernel as the kernel function in this study, and through the grid search method to seek the best c (penalty coefficient) and width parameter g (gamma). In addition, the “leave-one-out” cross-validation method was applied to obtain the highest accuracy, sensitivity and specificity. The area under the curve (AUC) of receiver-operating characteristic (ROC) and were applied to evaluate the predictive performance of each established model.

### Statistical analysis

Independent-sample t-test and chi-squared test were used to compare demographic data between MDD patients and HCs by SPSS 24.0 software (SPSS, Chicago, IL, USA). All statistical tests were two-tailed, and *p* < 0.05 were considered statistically significant. One-sample t-test was performed on z-score maps for each mask to demonstrate within-group FC spatial distribution of each seed for MDD patients and HCs within a brain mask (*p* < 0.05, uncorrected) [54, 55], which were also reported in several studies of FC analysis [56, 57]. Then the two-sample t-test was performed to assess significant differences of the whole brain FC in each region between MDD patients and HCs within the union mask of one-sample t-test results of both groups, by controlling for age, gender, years of education and the mean FD. The cluster-level multiple comparison correction was conducted using Gaussian random field (GRF) theory correction (voxel *p* value < 0.005; cluster *p* value < 0.006, 0.05/8, GRF corrected).

To further explore the relationship among the factors derived from the brain function, gut microbiota and clinical variables, spearman correlation coefficient was calculated among the FC values, gut microbiota (alpha and beta diversity, and relative abundance) and clinical variables (onset age of illness, number of episodes, duration of illness, 24-item HDRS scores and HAMA scores). The significant level was set as *p* < 0.05.

## Results

### Demographic and clinical characteristics

The demographic and clinical data of all study participants were presented in Table 1. Five patients with MDD and 2 HCs were excluded from further analyses because of excessive head motion during the image acquisition. Finally, 44 patients with MDD and 42 HCs were included in this analysis. No significant differences were found in age, gender, or education levels between the MDD and HCs group (all *p* > 0.05).

**Table 1:** Demographic and clinical data for patients with MDD and HCs.

	MDD	HCs	*p* value
	Mean (S.D.)	Mean (S.D.)	
Number of subjects	44	42	n/a
Age (years)	32.39 (13.26)	33.21 (13.05)	0.773*
Sex (male/female)	11/30	16 /28	0.191^a^
Education (years)	13.04 (2.57)	14.53 (4.74)	0.115*
Age at onset (years)	29.31 (13.98)	n/a	n/a
Number of episodes	1.85 (1.16)	n/a	n/a
24-item HDRS (score)	29.47 (6.85)	0–7	n/a
Duration of illness (months)	30.25 (34.34)	n/a	n/a
FD	0.050 (0.029)	0.049 (0.033)	0.893*

*MDD* major depressive disorder, *HCs* healthy controls, *HDRS* Hamilton Depression Rating Scale, *FD* framewise displacement for in-scanner head motion.

*The *p* values were obtained by Mann–Whitney *U* tests.

^a^The *p* value for sex distribution was obtained by chi-square test.

### FC changes of hippocampus and hippocampal subregions

Firstly, we have selected the left and right whole hippocampus separately as a single seed to conduct whole brain FC analysis, and found no difference between patients with MDD and HCs. The FC spatial distributions of left and right whole hippocampus in the MDD group were similar to those of the HCs (Fig. S1). Further, seed-based FC analyses of hippocampal subregions were conducted, as shown in Fig. 1B–E and Table 2. The FC spatial distributions of hippocampal subregions in the MDD group were similar to those of the HCs (Fig. S2). Compared with HCs group, the patients with MDD showed increased FC between the left CA2 and right hippocampus (mainly CA2 and CA3), left CA3 and right hippocampus (mainly CA2 and CA3), left DG and right hippocampus (mainly CA2 and CA3), and decreased FC between the right CA3 and bilateral PCC (Fig. 1B–E and Table 2).

**Table 2:** The areas of significantly different FC between the MDD patients and the HCs (voxel *p* < 0.005, cluster *p* < 0.006, GRF corrected).

Subregions	Location in the cerebrum	MNI coordinates			Peak *t* value	Cluster size (voxel numbers)
		*X*	*Y*	*Z*		
L CA2	R caudal hippocampus (mainly CA2 and CA3)	33	-33	0	4.48	64
L CA3	R caudal hippocampus (mainly CA2 and CA3)	33	-30	-3	5.16	68
L DG	R caudal hippocampus (mainly CA2 and CA3)	33	-33	0	5.33	79
R CA3	L PCC	-3	-48	30	-3.58	51

*FC* functional connectivity, *MDD* major depressive disorder, *HCs* healthy controls, *GRF* Gaussian random field, *CA* cornu ammonis, *PCC* posterior cingulate cortex, L (R) left (right) hemisphere.

### Analysis of gut microbiota in patients with MDD

**Fig. 2:**
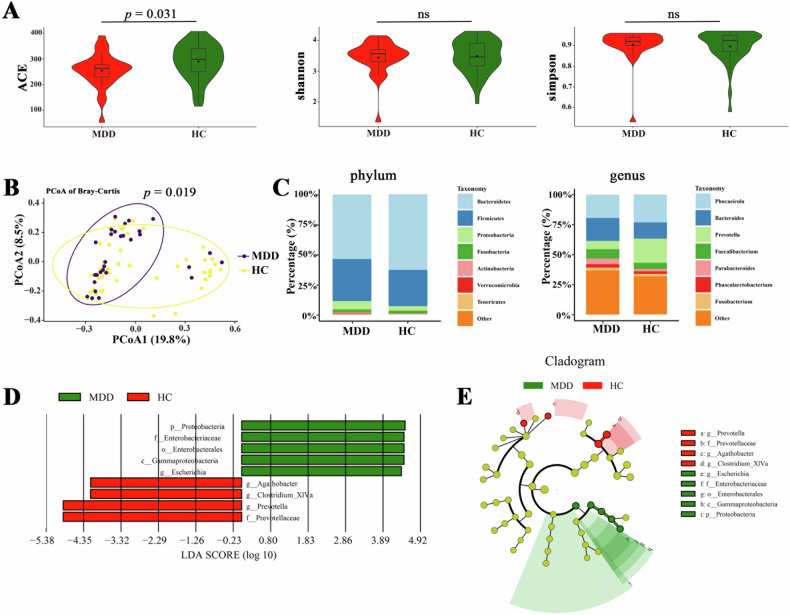
Gut microbial characteristics in MDD. **A** Microbial alpha-diversity indices (ACE, shannon and simpson) between MDD and HCs. **B** Principal coordinate analysis (PCoA) based on Bray–Curtis distances comparing the sample distribution between the two groups. The purple dots represent MDD, and the yellow dots represent HCs. **C** Microbial composition at the phylum and genus levels in MDD and HCs. **D** Linear discriminant analysis (LDA) scores derived from LEfSe analysis, showing the biomarker taxa (LDA scores (log10) > 2.0 and a significance of *p* < 0.05 determined by the Wilcoxon signed-rank test. Green and red colors represent an increase and decrease of abundance. **E** Cladogram generated from LEfSe analysis showing the relationship between taxon (the levels represent, from the inner to outer rings, phylum, class, order, family, and genus).

Alpha-diversity values including species richness indices (ACE) and species diversity indices (Shannon and Simpson) were compared between MDD and HC groups. We found that the ACE index was significant decreased (*p* = 0.031), whereas the indices of Shannon and Simpson were not significant alterations in patients with MDD relative to HCs (Fig. 2A). In addition, the beta diversity analysis further indicated that there was a significant difference in microbiota structural composition between the two groups (*p* = 0.019), and the PCoA of the Bray-Curtis distance metrics showed that the MDD and HC groups formed distinct clusters (Fig. 2B). The histogram of the microbial composition of the genus level showed that sequences from the MDD group were mainly assigned to *Phocaeicola* and *Bacteroides*, followed by *Prevotella* and *Faecalibacterium*; and sequences from HC group were mainly assigned to *Phocaeicola* and *Prevotella*, followed by *Bacteroides* and *Faecalibacterium* (Fig. 2C). By using the LEfSe differential analysis based on the all levels of species abundance, it was found that the MDD group was mainly enriched in the *Proteobacteria*, *Gammaproteobacteria*, *Enterobacterales*, *Enterobacteriaceae* and *Escherichia*; while HC group was mainly enriched in the *Prevotellaceae*, *Prevotella*, *Agathobacter*, and *Clostridium* (Fig. 2D, E).

### Correlation analyses

**Fig. 3:**
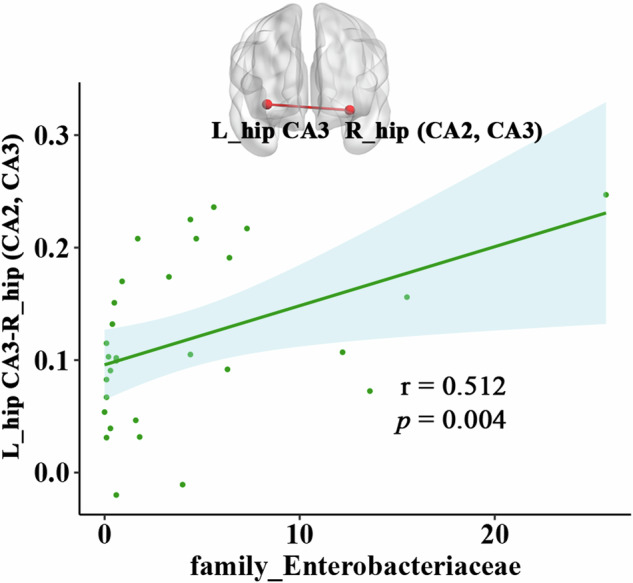
The correlation between the abnormal FC values of hippocampal subregions and relative abundance of family *Enterobacteriaceae* in MDD patients. FC functional connectivity, MDD major depressive disorder, hip hippocampus, CA cornu ammonis.

FC values of the left CA3- right caudal hippocampus (mainly CA2 and CA3) was positively correlated with the relative abundance of family *Enterobacteriaceae* (*r* = 0.512, *p* = 0.004) only in patients with MDD (Fig.3). In addition, illness duration was negatively correlated with genus *Prevotella* (*r* = −0.504, *p* = 0.044) in patients with MDD (Fig. S3). No significant correlation was found between abnormal FC value of hippocampal subregions and clinical variables (including onset age of illness, illness duration, number of episodes, 24-item HDRS score).

### SVM classification analysis

**Fig. 4:**
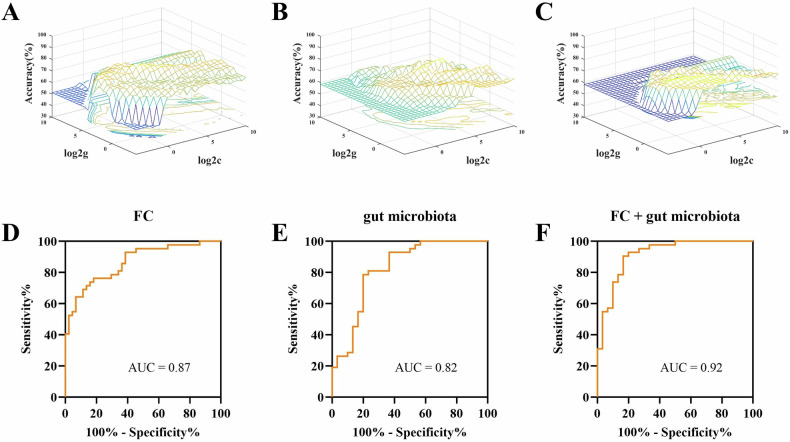
Visualization of classifications through support vector machine (SVM) using altered FC pattern of hippocampal subregion and relative abundance of gut microbiota. **A**–**C** Searching the optimal parameters of SVM models through the grid search method. **D**–**F** ROC curves assessing SVM performance. **A**, **D** FC as features; **B**, **E** gut microbiota as features; **C**, **F** combination of FC and gut microbiota as features. AUC area under the curve.

The hippocampal subregion FC values of altered brain regions and relative abundance of altered gut microbiota were used as features separately or together in the SVM models distinguishing the MDD patients and HCs. Fig. 4 and Table S1 show the results of the SVM classification between MDD patients and HCs. The AUC values of the classification models of altered hippocampal subregion FC values and altered relative abundance of gut microbiota were 0.87 and 0.82, respectively. Thus, when hippocampal subregion FC values and relative abundance of gut microbiota were considered in combination, the best discrimination was obtained (AUC = 0.92, accuracy = 80.56%, sensitivity = 90.48%, and specificity = 80.00%).

## Discussion

The main findings are as follows: (i) Increased FC was found between the bilateral hippocampus (mainly CA2, CA3 and DG); and decreased FC between the right hippocampal CA3 and bilateral PCC in MDD group; (ii) significant differences were showed in alpha-diversity (ACE index) and beta-diversity in MDD group compared to HC group; LEfSe analysis identified increased levels of proinflammatory bacteria (i.e., *Enterobacteriaceae*), and decreased short-chain fatty acids (SCFAs) producing-bacteria (i.e., *Prevotellaceae*, *Agathobacter*, and *Clostridium***)** in patients with MDD; (iii) FC values of the left hippocampal CA3-right hippocampus (CA2 and CA3) was positively correlated with the relative abundance of family *Enterobacteriaceae* in MDD patients and (iv) Combined hippocampal subregions’ FC and gut microbiota features could achieve higher accuracies (AUC = 0.92, accuracy = 80.56%, sensitivity = 90.48% and specificity = 80.00%) than any single feature method. To the best of our knowledge, this study is the first to investigate the association between the FC of hippocampal subregions and microbial composition in unmedicated non-late-life adult patients with MDD, which may help us reveal the biological mechanisms underlying MDD from the new perspective of gut-brain axis interactions and further find possible treatment target for MDD. In addition, the abnormal FC patterns of hippocampal subregions and relative abundance of gut microbiota might be biomarkers distinguishing MDD and HCs.

In current study, we found increased FC between the left hippocampal CA2 and right hippocampus (mainly CA2 and CA3), between left hippocampal CA3 and right hippocampus (mainly CA2 and CA3), and between left DG and right hippocampus (mainly CA2 and CA3) in unmedicated patients with MDD, suggesting abnormal bilateral hippocampus connectivity, especially in the CA2, CA3 and DG subregions in MDD. The caudal hippocampus, such as CA2 and CA3, is implicated in cognitive functions (including learning, associative memory and spatial navigation), abnormal FC of the caudal hippocampus might contribute to declarative memory impairments or to impairments in episodic memory and spatial span performance in MDD [18, 58, 59]. A fMRI study based on graph theory method found aberrant FC strength between bilateral caudal hippocampus in first-episode drug-naïve patients with MDD [60], which suggested that hippocampal function had already been abnormal in the early stages of MDD. Task-based fMRI meta-analysis revealed hyperactivity in the hippocampus in adults with MDD during cognitive and emotional processing tasks [61, 62]. Another study elucidated that abnormal brain activity in the CA3 and DG was associated with depressive symptoms in patients with MDD during performing cognitive task [63]. Results from several animal studies suggested that the neurogenesis in the DG could be suppressed by chronic stress [64, 65]. In addition, recent structural morphometric studies exhibited reduced hippocampal subregion volumes in the CA2, CA3 and DG in patients with MDD [66–68]. Studies on postmortem brain issue provided evidence of alterations in cellular architecture and/or morphology in hippocampus in MDD patients, including reduced hippocampal volume, decreased spine density in hippocampal CA3 pyramidal neurons numbers and glia density, and decreased vascularization of the neurogenic niche [12, 69]. Thus, we speculated that the increased FC between the bilateral hippocampus might be a compensatory response to the structural deficits of hippocampus in MDD, which might be associate to cognitive impairment of MDD.

The gut microbiome is a crucial node for understanding the mechanisms of MDD, which has been suggested to influence cognitive functioning and mood through neural signaling, metabolic and immune-mediated mechanisms [70, 71]. In this study, increased relative abundance of proinflammatory bacteria (i.e., *Enterobacteriaceae*), and decreased relative abundance of SCFAs producing-bacteria (i.e., *Prevotellaceae* and *Lachnospiraceae***)** were found in patients with MDD. These findings were gently consistent with previous systematic reviews and meta-analyses [9, 72, 73]. More importantly, the relative abundance of *Enterobacteriaceae* were positively correlated with the increased FC between left hippocampal CA3 and right caudal hippocampus in patients with MDD. The *Enterobacteriaceae*, is gram-negative bacteria that colonize in normal gut flora and help maintain intestinal homeostasis [74]. Overgrowth of *Enterobacteriaceae* could result in gut inflammation and disruption of the gut barrier, which in turn favors promoting low-grade systemic inflammation through the bacterial translocation and bacterial products into the systemic circulation, and further acrossing the blood-brain barrier (BBB), thereby damaging cognitive function in patients with MDD [75–77]. Several animal studies demonstrated that peripheral injection of *Enterobacteriaceae* could promote higher levels of pro-inflammatory cytokine and microglial cell marker gene expression [78–80] and lead to decreased expression of the BDNF [81, 82] in the hippocampus, then induced learning and memory impairments. Taken together, the increase of proinflammatory bacteria (i.e., *Enterobacteriaceae*) would lead to abnormal FC between the left hippocampus and right hippocampus, which may cause by hippocampal inflammation by the gut barrier disruption in MDD. On the other hand, elevated low-grade inflammatory activity in depressed individuals also could enhance damage to the gut barrier through GBA that predispose them to altered colonization by gut flora [7, 42].

In addition, patients with MDD showed decreased FC between the right CA3 of hippocampus and the bilateral PCC. The PCC is a core node of the default mode network (DMN), which is involved in affective processing and internally-directed cognition [83–85]. A structural meta-analysis identified abnormal cortical thickness in the PCC of MDD patients [86]. Previous rs-fMRI studies found decreased FC between the hippocampal CA and DMN (including PCC) in patients with MDD [87, 88]. Another recent study revealed that higher connectivity between the left hippocampus and PCC has potential to predict better outcomes of treatment, and the connectivity was correlated with depressive symptom, in patients with MDD [29]. Furthermore, a task-based fMRI study also found abnormal activation in the PCC during rumination induction task in patients with MDD [89]. Therefore, our findings of disrupted FC between the hippocampal subregions and PCC in MDD might be related to depressive symptoms in MDD.

Our findings revealed that the index of alpha-diversity (ACE) was decreased in MDD patients, suggesting that a lower diversity of gut microbiota in patients with MDD than HCs. Likewise, beta-diversity analysis displayed significant differences in the gut microbiota composition between the two groups. These findings were also reported in previous systematic reviews and meta-analyses [9, 72, 73]. Moreover, our findings also found the illness duration was negatively correlated with relative abundance of SCFAs-producing bacteria (i.e., *Prevotella*) in MDD patients, which suggests that a decrease in SCFAs-producing bacteria may affect course of depression in patients with MDD. The genus *Prevotella* belongs to the family *Prevotellaceae*, which is generally related to the production of SCFAs and significantly contributes to maintain the stability of intestinal environment [90–92]. Reduced levels of these gut bacteria may result in aberrant cognitive, immunological and inflammatory responses [93, 94], which may contribute to the pathophysiology of MDD.

In addition, SVM model showed that combined features of altered hippocampal subregions’ FC and gut microbiota level exhibited highest AUC value (AUC = 0.92) than any single feature method to classify MDD and HCs, indicating that the abnormal FC patterns of hippocampal subregions and the level of gut microbiota have better classification performance and diagnostic value in distinguishing MDD and HCs. Previously, SVM analysis of diagnostic classifiers for MDD were mostly based on gut microbiota or neuroimaging markers single-modal [45, 95], and the prediction accuracy was mostly lower than our performance. Our results suggested that the multimodal combination feature might be a better way when distinguish MDD from HCs.

### Limitations

Several limitations of this study should be considered. First, a relatively small sample of patients were recruited from a single center, which may cause selection bias. Second, due to the small size of hippocampal subfields relative to the voxel size acquired for fMRI and limited resolution fMRI, it is challenging to delineate its structure, distinguish between the subregions, and confirm whether the hippocampus signal is contaminated by the adjacent structure. Future studies applying fMRI with higher resolution would provide a deeper understanding of their potentially different roles in MDD. Third, this study was a preliminary cross-sectional study. Although we detected correlations between brain function alterations and the gut microbiota composition as well as diversity in MDD, we could not identify the causal relationship between them. Fourth, results of correlations analyses between brain FC values and gut microbiota are considered exploratory as they were not corrected for multiple comparisons. Thus, future longitudinal studies with large sample size might help to identify the causal relationship between the gut microbiota composition and brain function alterations. In addition, the metabolic and inflammatory alteration of gut microbiota should be further investigated to shed light on the possible potential mechanisms. Furthermore, although all participants were recruited from the same site and were used very strict exclusion criteria to control the potential effect of factors, such as taking oral probiotics or yogurt, we could not eliminate all possible confounders affecting gut microbiota composition.

## Conclusions

In conclusion, our study found that abnormal FC between the bilateral hippocampus (mainly the CA2 and CA3), and between the hippocampal CA3 and PCC in MDD. Increased levels of proinflammatory bacteria (i.e., *Enterobacteriaceae*), and decreased SCFAs producing-bacteria (i.e., *Prevotellaceae, Agathobacter, and Clostridium***)** were found in patients with MDD. Moreover, abnormal FC between the bilateral hippocampus was associated with increased relative abundance of proinflammatory bacteria. In addition, the abnormal FC patterns of hippocampal subregions and relative abundance of gut microbiota might be biomarkers distinguishing MDD and HCs. These findings may provide insights into the potential role of gut microbiota in the underlying neuropathology of MDD patients, and may have important clinical implications in targeting the gut microbiota as a treatment target of MDD patients.

### Supplementary information

Supplementary materials

## Data Availability

The sequencing data supporting the conclusions of this article are available in the NCBI Bioproject repository, PRJNA936479.
