# Supplementary material for: Gut proinflammatory bacteria is associated with abnormal functional connectivity of hippocampus in unmedicated patients with major depressive disorder
Source: Transl Psychiatry. 2024 Jul 16;14:292. doi: 10.1038/s41398-024-03012-9 (PMC11253007; doi:10.1038/s41398-024-03012-9)
Supplement: Supplementary file 1 — Supplementary materials [file 41398_2024_3012_MOESM1_ESM.docx]

**Supplementary materials**

**Supplementary method**

**1. FC analyses of bilateral hippocampal subregions**

Seed-based FC analyses were also conducted for the bilateral hippocampal subregions (7 seeds in total including bilateral CA1, bilateral CA2, bilateral CA3, bilateral DG, bilateral EC, bilateral HATA, and bilateral Subc) using the DPABI toolbox. The cluster-level multiple comparison correction was conducted using Gaussian random field (GRF) theory correction (voxel *p* value < 0.007; cluster *p* value < 0.007, 0.05/7, GRF corrected).

**Supplementary results**

**2.1.** **FC analyses of bilateral hippocampal subregions**

We have repeated analyses using bilateral seed regions for each of the 7 hippocampal subregions, the results showed that decreased FC between the bilateral CA2 and bilateral PCC, between bilateral CA3 and bilateral PCC in MDD patients compared to HCs, as shown in the Supplementary materials (Fig.S4 and Table S2).

**2.2. FC analyses of hippocampal subregions with** **group-level tests correcting across the whole brain**

Moreover, we have repeated group-level tests correcting across the whole brain, and the results showed that increased FC between the left CA2 and right hippocampus (mainly CA2 and CA3), left DG and right hippocampus (mainly CA2 and CA3), and decreased FC between the right CA3 and bilateral PCC (Fig.S5 and Table S3), which generally consistent with our original findings.

**2.3. The volume of each of the hippocampal subregions**

In addition, the results of volume of each of the hippocampal subregions were presented in supplementary materials, and there has no differences in hippocampal subregion volumes between MDD patients and HCs (all *p >* 0.007[0.05/7], Bonferroni corrected), as shown in Table S4.


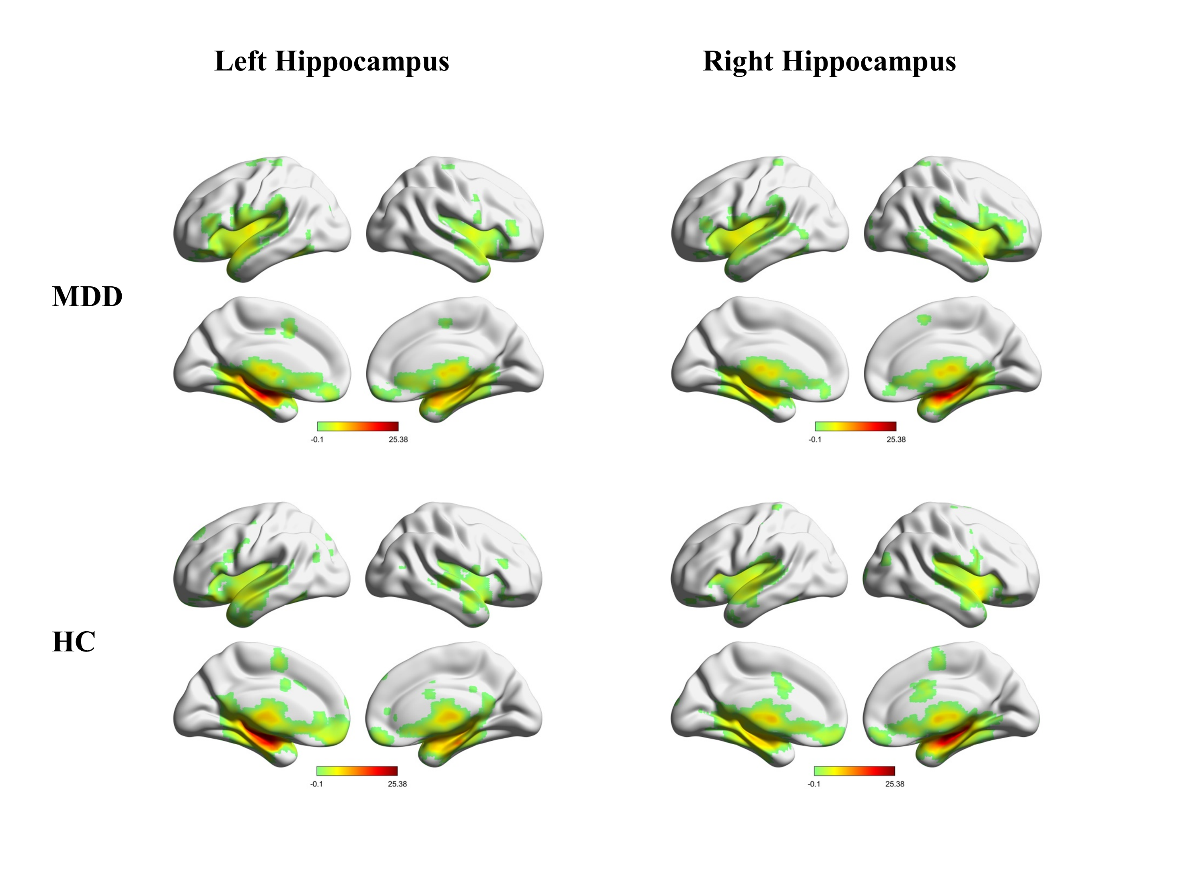


**Fig. S1.** The FC patterns of the left and right whole hippocampus in the MDD patients and HCs (p < 0.05, uncorrected). The color bar represents functional connection. FC, functional connectivity; MDD, major depressive disorder; HCs, healthy controls.


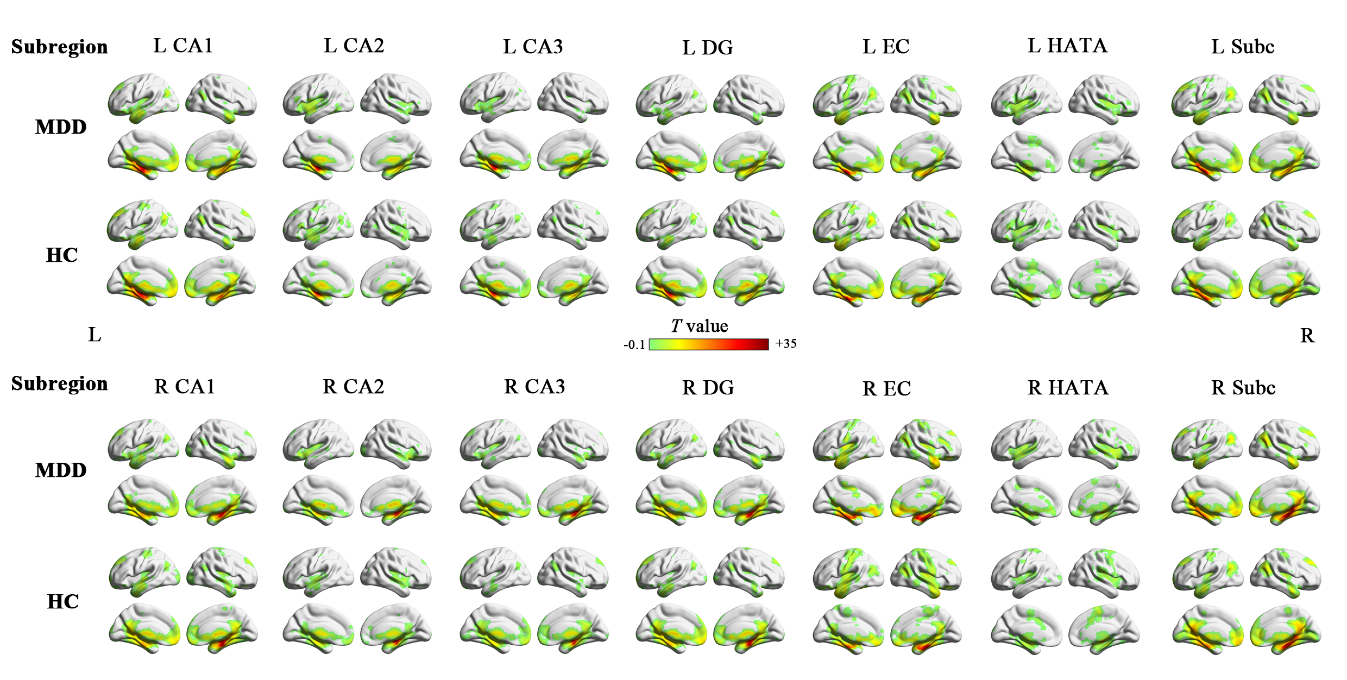


**Fig. S2.** The FC patterns of the hippocampal subregions in the MDD patients and HCs (p < 0.05, uncorrected). The color bar represents functional connection. FC, functional connectivity; MDD, major depressive disorder; HCs, healthy controls. CA, cornu ammonis, DG, dentate gyrus; EC, entorhinal cortex; HATA, hippocampal–amygdaloid transition area; Subc, subiculum. L, left; R, right.


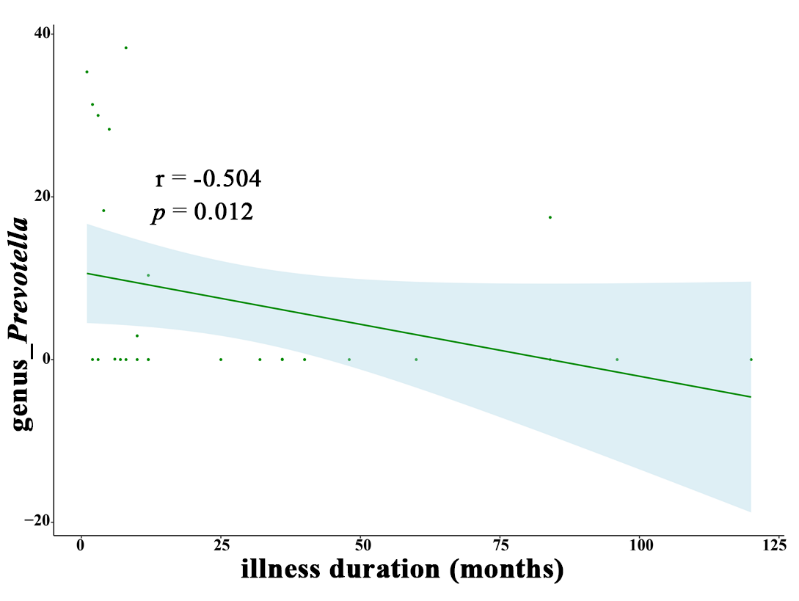


**Fig. S3.** The correlations between the illness duration and relative abundance of genus *Prevotella* in MDD patients.


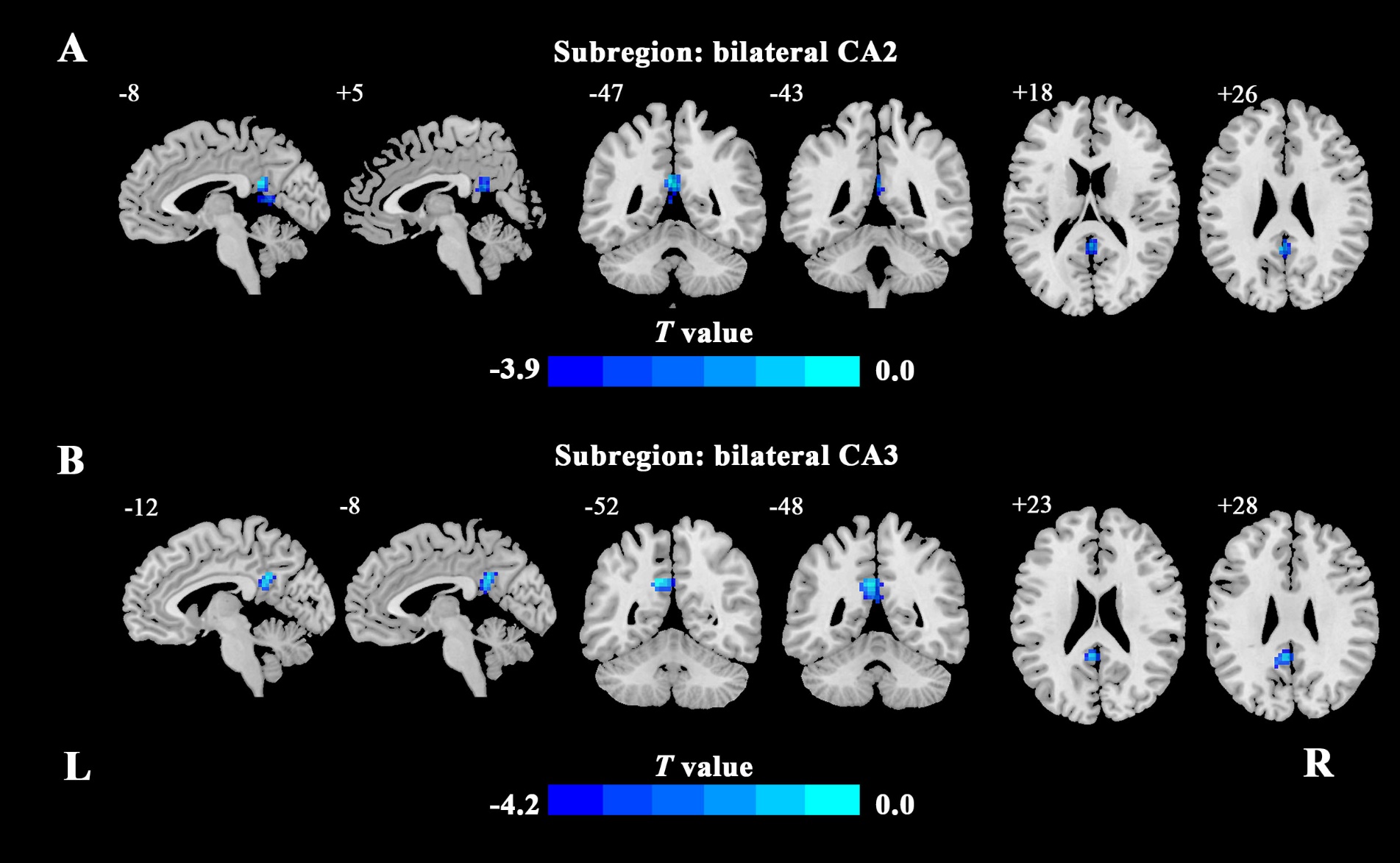


**Fig.S4.** The bilateral hippocampal subregions and significant FC differences between the patients with MDD and HCs. (A-B) Significant FC differences between the patients with MDD and HCs for hippocampal subregion, respectively (voxel *p* < 0.005, cluster *p* < 0.007, GRF corrected). The color bar indicates the *t* values from two-sample *t*-test analysis. FC, functional connectivity; GRF, Gaussian random field; CA, cornu ammonis; PCC, posterior cingulate cortex; L (R), left (right) hemisphere.


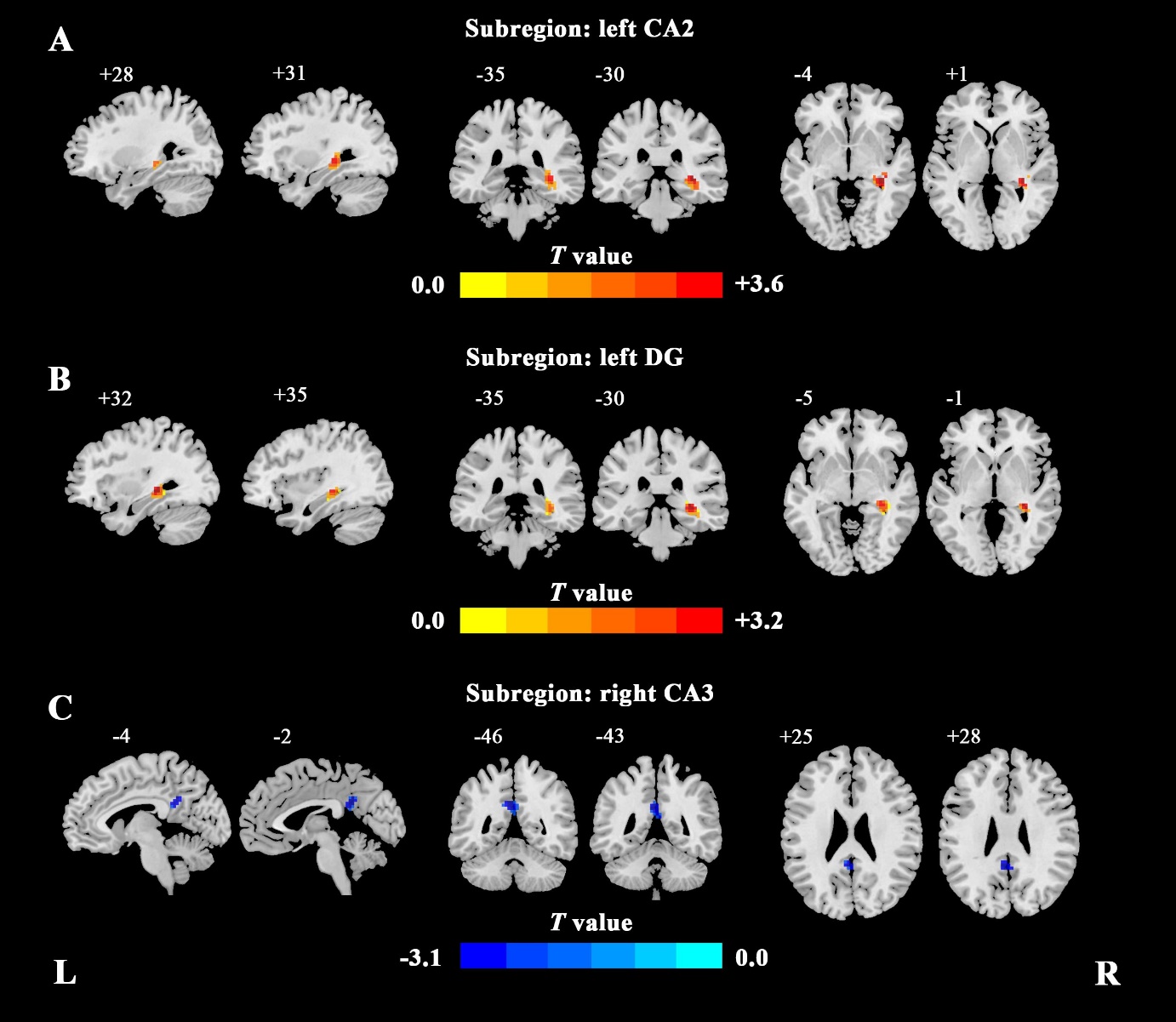


**Fig.S5.** The hippocampal subregions and significant FC differences with group-level tests correcting across the whole brain between the patients with MDD and HCs. (A-C) Significant FC differences between the patients with MDD and HCs for hippocampal subregion, respectively (voxel *p* < 0.005, cluster *p* < 0.007, GRF corrected). The color bar indicates the *t* values from two-sample *t*-test analysis. FC, functional connectivity; GRF, Gaussian random field; CA, cornu ammonis; PCC, posterior cingulate cortex; L (R), left (right) hemisphere.

**Table S1.** SVM classification performance of altered FC patterns of hippocampal subregions and relative abundance of gut microbiota singly and combinedly

|  | FC | gut microbiota | FC+ gut microbiota |
| --- | --- | --- | --- |
| Accuracy (%) | 83.72 | 75.00 | 80.56 |
| Sensitivity (%) | 76.19 | 78.57 | 90.48 |
| Specificity (%) | 81.82 | 80.00 | 80.00 |
| AUC | 0.87 | 0.82 | 0.92 |

**Table S2.** The areas of significantly different FC of bilateral hippocampal subregions between the MDD patients and HCs (voxel *p* < 0.005, cluster *p* < 0.007, GRF corrected)

| Subregions | Location in the cerebrum | MNI coordinates | | | Peak *t* value | Cluster size  (voxel numbers) |
| --- | --- | --- | --- | --- | --- | --- |
|  |  | X | Y | Z |  |  |
| Bilateral CA2 | bilateral PCC | 0 | -45 | 21 | -4.21 | 69 |
| Bilateral CA3 | bilateral PCC | -6 | -48 | 30 | -3.88 | 71 |

Abbreviations: FC, functional connectivity; MDD, major depressive disorder; HCs, healthy controls; GRF, Gaussian random field; CA, cornu ammonis; PCC, posterior cingulate cortex; L (R), left (right) hemisphere.

**Table S3.** The areas of significantly different FC with group-level tests correcting across the whole brain between the patients with MDD and HCs (voxel *p* < 0.005, cluster *p* < 0.007, GRF corrected)

| Subregions | Location in the cerebrum | MNI coordinates | | | Peak *t* value | Cluster size (voxel numbers) |
| --- | --- | --- | --- | --- | --- | --- |
|  |  | X | Y | Z |  |  |
| L CA2 | R caudal hippocampus (mainly CA2 and CA3) | 33 | -33 | 0 | 3.62 | 65 |
| L DG | R caudal hippocampus (mainly CA2 and CA3) | 33 | -33 | 0 | 3.28 | 68 |
| R CA3 | L PCC | -3 | -48 | 30 | -3.13 | 43 |

Abbreviations: FC, functional connectivity; MDD, major depressive disorder; HCs, healthy controls; GRF, Gaussian random field; CA, cornu ammonis; PCC, posterior cingulate cortex; L (R), left (right) hemisphere.

**Table S4.** The volume of each of the hippocampal subregions in MDD patients and HCs (all *p >* 0.007[0.05/7], Bonferroni corrected)

| Subregion | Volume (Mean±SD) cm^3^ | | *p* value | *t* value |
| --- | --- | --- | --- | --- |
|  | MDD | HC |  |  |
| CA1_L | 2.87±0.23 | 2.80±0.20 | 0.117 | 1.582 |
| CA1_R | 2.73±0.22 | 2.65±0.18 | 0.075 | 1.805 |
| CA2_L | 0.88±0.08 | 0.85±0.06 | 0.107 | 1.631 |
| CA2_R | 1.19±0.10 | 1.15±0.09 | 0.040 | 2.090 |
| CA3_L | 1.29±0.11 | 1.25±0.09 | 0.106 | 1.636 |
| CA3_R | 1.38±0.11 | 1.34±0.10 | 0.067 | 1.859 |
| DG_L | 1.93±0.16 | 1.89±0.13 | 0.153 | 1.441 |
| DG_R | 1.83±0.14 | 1.77±0.13 | 0.083 | 1.756 |
| EC_L | 2.94±0.28 | 2.79±0.21 | 0.009 | 2.670 |
| EC_R | 3.13±0.30 | 3.07±0.28 | 0.310 | 1.022 |
| HATA_L | 0.24±0.02 | 0.23±0.02 | 0.017 | 2.426 |
| HATA_R | 0.22±0.02 | 0.22±0.02 | 0.136 | 1.504 |
| Subc_L | 4.05±0.29 | 3.97±0.27 | 0.191 | 1.317 |
| Subc_R | 3.87±0.29 | 3.81±0.26 | 0.255 | 1.147 |

Abbreviations: MDD, major depressive disorder; HCs, healthy controls; CA, cornu ammonis; DG, dentate gyrus; EC, entorhinal cortex; HATA, hippocampal–amygdaloid transition area; Subc, subiculum; L (R), left (right) hemisphere.
